# A Pilot Study on a Reliable and Accessible Approach to Remote Mental Health Assessment: Lessons from Italian Pregnant Women During the COVID-19 Pandemic

**DOI:** 10.3390/healthcare13212762

**Published:** 2025-10-30

**Authors:** Chiara Colliva, Veronica Rivi, Pierfrancesco Sarti, Alice Ferretti, Giulia Ganassi, Lorenzo Aguzzoli, Johanna Maria Catharina Blom

**Affiliations:** 1 Department of Biomedical, Metabolic and Neural Sciences, University of Modena and Reggio Emilia, 41125 Modena, Italy; chiara.colliva@unimore.it (C.C.); pierfrancesco.sarti@unimore.it (P.S.); 2 Department of Life Sciences, University of Modena and Reggio Emilia, 41125 Modena, Italy; 3 Obstetrics and Gynaecology Unit, Arcispedale “Santa Maria Nuova” AUSL—IRCCS, 42123 Reggio Emilia, Italy; alice.ferretti@ausl.re.it (A.F.); lorenzo.aguzzoli@ausl.re.it (L.A.); 4 San Michele Private Outpatient Clinic, 42121 Reggio Emilia, Italy; giulia.ganassi@gmail.com; 5 Centre of Neuroscience and Neurotechnology, University of Modena and Reggio Emilia, 41125 Modena, Italy

**Keywords:** remote monitoring, perinatal care, pregnancy, maternal mental health, risk stratification, targeting health

## Abstract

**Objective**: This pilot study assessed the psychological and physical impact of the COVID-19 pandemic on postpartum women that gave birth during the pandemic, and evaluated the feasibility of remote monitoring for maternal mental health. The study also proposes a conceptual framework to strengthen remote maternal care in future public health emergencies. **Methods**: Conducted between 2020 and 2021 in Reggio Emilia, one of Italy’s ten hardest-hit provinces during the early COVID-19 outbreak, this study enrolled 21 pregnant women (10 COVID-19-positive at delivery, 11 COVID-19-negative controls). Psychological and physical health were assessed using validated instruments: the Beck Depression Inventory (BDI) and Edinburgh Postnatal Depression Scale (EPDS) for depression, the State-Trait Anxiety Inventory (STAI) for anxiety, the Impact of Event Scale–Revised (IES-R) for trauma-related stress, and the SF-36 for physical functioning. Additional measures included breastfeeding experience and resilience. Remote assessments were conducted between 6 and 12 months postpartum to evaluate psychological recovery and satisfaction with perinatal care. C test was used to compare the two groups of women. **Results**: COVID-19-positive women reported significantly higher depressive symptoms (BDI: 13.50 ± 8.14 vs. 6.73 ± 4.73; U = 27, *p* = 0.048), and elevated state anxiety levels (STAI-S: 41.60 ± 10.23 vs. 33.64 ± 10.15; U = 27, *p* = 0.048) compared to controls. Post-traumatic stress symptoms were also higher among COVID-positive participants (IES-R total: 41.10 ± 19.33 vs. 30.64 ± 7.99; U = 24.5, *p* = 0.029). No significant differences emerged in EPDS or trait anxiety scores. **Conclusions**: Remote data collection proved feasible for postpartum women during the pandemic and highlighted elevated depressive, anxiety, and trauma-related symptoms in COVID-19-positive mothers. These findings support the development of flexible digital care frameworks for maternal well-being in crises. The introduction of the “10 Gold Rules for Remote Maternal Healthcare in Critical Situations” offers a forward-looking, expert-informed conceptual framework to guide the development of scalable, trust-based digital care models that go beyond monitoring to include proactive, patient-centred support.

## 1. Introduction

Pregnancy and perinatal periods involve profound physiological and psychological changes, requiring significant adaptation and resilience [1,2,3]. During this transition, women face rapid hormonal shifts, sleep disruption, and emotional variability, while simultaneously adjusting to new maternal roles and responsibilities. These changes are typically accompanied by increased psychological vulnerability, making the perinatal period a crucial window for both prevention and early support. Understanding the dynamic balance between biological, emotional, and contextual factors during this stage is essential to promote maternal and infant well-being.

The COVID-19 pandemic dramatically amplified these challenges, adding uncertainty, isolation, and fear into an already delicate life phase [4,5,6,7]. Pregnant and postpartum women were exposed to pervasive stressors, including disruptions in prenatal and perinatal care, restrictions on partner presence during delivery, and heightened concern about infection risk and foetal outcomes [8,9,10,11,12]. For those who tested positive for COVID-19, the psychological burden was severe, combining the usual anxieties associated with childbirth with the fear of a potentially life-threatening illness [5,6]. Even uninfected women experienced significant emotional distress, with increased rates of anxiety, depression, and trauma-related symptoms linked to social isolation, healthcare limitations, and economic insecurity [7]. As a result, what is usually an intimate and supportive experience—labour and delivery—was characterised by limitations, loneliness, and heightened emotional stress [12].

These unprecedented circumstances highlighted the urgent need for adaptable and resilient models of maternal care [13]. Traditional healthcare systems, optimised for stable conditions, were often unable to meet the complex psychological and social needs of perinatal women during prolonged emergencies [14,15]. Guidelines often prioritise resource allocation and containment, but the pandemic emphasised the need for a more nuanced approach that balances public health measures with economic stability, social cohesion, and mental health support [16]. In this context, remote assessment and telemedicine approaches emerged as essential tools to maintain continuity of care, support emotional well-being, and enable the early identification of at-risk individuals [17].

Against this backdrop, the present exploratory pilot study investigates the feasibility of remote data collection to monitor the mental health of postpartum women that were pregnant during the COVID-19 pandemic, comparing psychological outcomes between women who tested positive for COVID-19 at delivery and those who did not. To our knowledge, this is the first Italian pilot study to apply a fully remote, non-interventional monitoring protocol for assessing maternal psychological and physical well-being in the postpartum period during a public health emergency. By combining standardised self-report tools with a flexible, telematic model, the study introduces a novel, accessible framework for collecting high-quality mental health data while minimising participant burden and infection risk.

Importantly, the present study was conducted in Reggio Emilia, a province that ranked among the ten hardest-hit areas in Italy during the initial phase of the COVID-19 pandemic, reporting 63 confirmed COVID-19 cases per 10,000 inhabitants and ranking seventh in infection rate across Italian provinces (https://www.ausl.re.it/anno-2021-covid-dati, accessed on 30 October 2025). Conducted in this high-risk context, the study aims to evaluate the feasibility of remote, patient-centred monitoring of postpartum mental health, and to propose a scalable methodological framework that could facilitate early identification and stratification of women at higher psychological risk during future public health emergencies.

## 2. Materials and Methods

### 2.1. Study Design

This exploratory pilot study aimed to propose the feasibility of remote data collection and identify preliminary trends in maternal mental health during the COVID-19 pandemic. Two distinct cohorts were enrolled from May 2020 to the end of 2021: women who remained COVID-19-negative during pregnancy (Group 1, N = 11, aged 28–40 years) and women who tested positive for COVID-19 at the time of labour (Group 2, N = 10, aged 31–44 years). The COVID-positive group included women diagnosed with confirmed SARS-CoV-2 infection via PCR testing during hospitalisation for childbirth. The severity of COVID-19 symptoms among participants ranged from asymptomatic or mild; importantly, none of the women required intensive care or mechanical ventilation and analyses by severity were not conducted due to homogeneity of symptom presentation. None of the pregnancies in either group was classified as high-risk, and there were no obstetric emergencies or complex deliveries. Importantly, the study did not target women with a clinical diagnosis of postpartum depression or other psychiatric disorders. Participants met the inclusion criteria of confirmed pregnancy during the pandemic, completion of relevant health and psychological assessments, and access to standard clinical care and support services as needed. Exclusion criteria comprised preterm births (<38 weeks of gestation), pre-existing psychiatric diagnoses, unrelated medical complications, and incomplete data.

The study protocol was approved by the Ethics Committee of Modena (Prot. PRE BIO CE n. 10136, 23 March 2020), and all participants provided written informed consent.

### 2.2. Data Collection

Data collection was conducted remotely between 6 and 12 months postpartum to capture changes in participants’ psychological and physical well-being during this period.

Eligible women were identified through hospital delivery records (COVID-19-positive group) at the Obstetrics and Gynaecology Unit of Arcispedale “Santa Maria Nuova” (Reggio Emilia, Italy) or during routine outpatient visits (non-COVID group).

After being individually contacted by the research team, participants received detailed study information and provided written informed consent before completing the assessments. Data were collected between May 2020 and December 2021, encompassing both psychological and physical health domains.

Each woman was invited to complete a battery of standardised, self-report questionnaires (described in Section 2.3) to evaluate mental health, physical health, and perceived challenges associated with pregnancy, breastfeeding, and the pandemic. All assessments were conducted in digital format via encrypted links, allowing participants to respond autonomously at home within a flexible time window.

No structured psychological or clinical intervention was provided in conjunction with data collection, which was observational in nature. Reminder messages and technical support were offered to maximise participation. All 21 women completed the full assessment set, resulting in no attrition or missing data.

### 2.3. Tools and Questionnaires

A battery of standardised and validated self-report instruments assessing depressive and anxiety symptoms, trauma-related stress, and physical functioning. All measures have been widely used in perinatal research and are suitable for remote administration.

- Edinburgh Postnatal Depression Scale (EPDS, pre- and postpartum versions) [18]: A 10-item self-report questionnaire designed to assess the presence and severity of depressive symptoms in women during the postpartum period. Each item is scored on a 4-point scale, ranging from 0 to 3. Total scores range from 0 to 30, with scores ≥ 13 indicating probable postnatal depression requiring clinical attention.
- State-Trait Anxiety Inventory (STAI-Y) [19]: Measures state anxiety (temporary feelings influenced by current stressors) and trait anxiety (stable tendencies to perceive situations as threatening). This inventory included 40 items, with 20 items dedicated to each component, rated on a 4-point Likert scale. Although no formal diagnostic thresholds exist, scores ≥ 40 are commonly interpreted as indicative of clinically significant levels of anxiety.
- Beck Depression Inventory (BDI) [20]: A 21-item self-report that measured depressive symptoms over the previous two weeks. Total scores range from 0 to 63 and are categorised as follows: 0–13 = minimal, 14–19 = mild, 20–28 = moderate, and ≥29 = severe depression. A cut-off score of ≥14 is typically used to indicate clinically relevant depressive symptoms.
- Beck Anxiety Inventory (BAI) [21]: A 21-item self-report scale measuring the intensity of anxiety symptoms such as nervousness or dizziness, scored on a 4-point scale (0–3). Total scores range from 0 to 63, with 0–7 = minimal, 8–15 = mild, 16–25 = moderate, and ≥26 = severe anxiety. A cut-off score of ≥16 is generally considered clinically significant.
- Short Form Health Survey (SF-36) [22]: A multidimensional instrument measuring health-related quality of life across 8 domains: physical functioning, role limitations due to physical problems, role limitations due to emotional problems, vitality/fatigue, emotional well-being, social functioning, pain, and general health. Each subscale is scored from 0 to 100, with higher scores indicating better health status. Although no standardised clinical cut-offs exist, scores below 60 on key domains are typically interpreted as indicative of impaired functioning or reduced quality of life.
- Impact of Event Scale-Revised (IES-R) [23]: A 22-item self-report measure of trauma-related distress, covering intrusion, avoidance, and hyperarousal. Items are rated from 0 (not at all) to 4 (extremely), with a total score ranging from 0 to 88. A total score ≥ 33 suggests clinically concerning post-traumatic stress symptoms, while subscale scores ≥ 1.5–2.0 may indicate significant distress in individual domains. All these self-administered tests were conducted remotely to accommodate the delicate postpartum period and minimise participant burden. The study’s telematic approach ensured accessibility while maintaining the accuracy and integrity of the collected data. Participants also provided self-reports regarding their breastfeeding experiences and challenges, including perceived benefits, clinician support, and overall difficulty.

This comprehensive approach allowed for the evaluation of multiple dimensions of health and well-being in the context of pregnancy, breastfeeding, and pandemic-related stressors.

No imputation methods were applied, and only complete case data were used in the analysis. Nonetheless, the small sample size and absence of imputation warrant caution when generalising findings. To avoid inflating the scope of our findings, we clearly define the limits of the observational model and adjust our interpretations accordingly.

### 2.4. Statistical Analysis

Descriptive statistics (means, standard deviations, and ranges) were used to summarise demographic, mental health, and physical health measures. A power analysis was conducted using G*Power 3.1. Given the sample size (N = 21; group sizes = 10 and 11), the study had approximately 74% power to detect a large between-group effect, at α = 0.05.

To study group differences, first, we analysed our data for normality assumption using the Kolmogorov–Smirnov one-sample test for normality (K-S distance and P): our data did not display a normal distribution. Thus, data were analysed using the Mann–Whitney U Test. All tests were defined as significant at *p* < 0.05. Data were presented as mean ± standard error (SEM). All statistical analyses were performed using SPSS v. 26.0 (IBM Corp., Armonk, NY, USA), while graphs were generated using GraphPad Prism v. 9.00e for MAC^®^ (GraphPad Software, Inc., La Jolla, CA, USA).

## 3. Results

### 3.1. Clinical and Anamnestic Variables Collected from the Control and the Study Groups

Starting at the time of hospital discharge, the examination of clinical and anamnestic variables collected remotely from the control group (pregnant women without COVID-19 infection) and the study group (pregnant women who contracted COVID-19) demonstrates substantial comparability across key demographic and baseline factors (Table 1). Both groups had similar average maternal ages (33.0 years in controls vs. 35.1 years in COVID-positive), comparable years of education (14.36 vs. 13.60), and closely matched family sizes (2.5 vs. 3.11 members). Partners’ age and education levels also showed no meaningful differences, supporting demographic homogeneity between groups. This similarity in baseline characteristics strengthens the validity of subsequent comparisons of psychological and physical health outcomes, as it reduces confounding by sociodemographic factors. Moreover, the prevalence of key clinical history variables such as first pregnancy status and anxiety during pregnancy was broadly aligned, further supporting group comparability.

While minor variations existed—such as a slightly higher proportion of first-time pregnancies and post-pregnancy anxiety in the COVID-positive group—these did not appear substantial enough to undermine group equivalence. Overall, the consistent clinical and anamnestic profiles between groups provide a sound basis for attributing observed differences in mental health outcomes primarily to COVID-19 infection status rather than underlying demographic or historical disparities. Building upon the established comparability of clinical and anamnestic data between the control and COVID-19-positive groups, we now present a detailed analysis of core outcome domains.

### 3.2. Mental Health Outcomes

**Figure 1:**
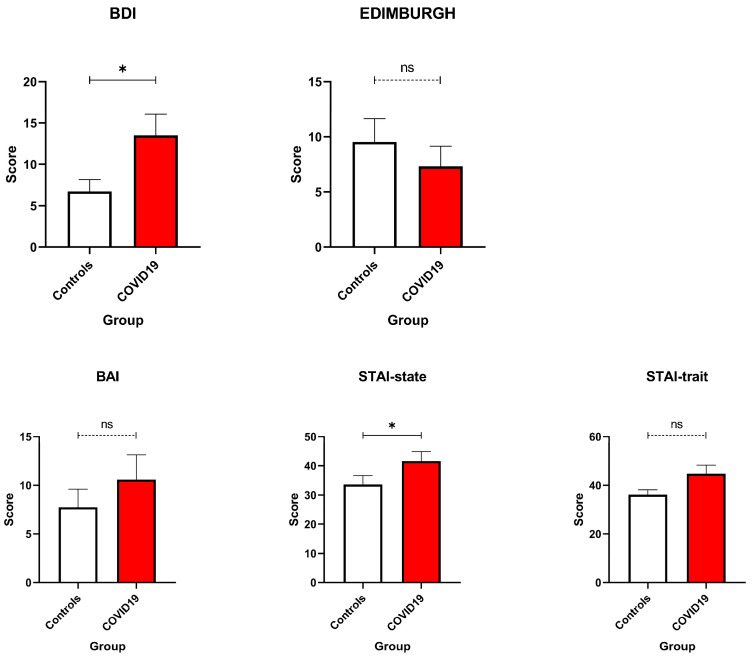
Anxiety and Depression Scales. BDI, BAI, STAI state, and STAI trait were compared between COVID-19 pregnant women (red—N = 10) and the control ones (white—N = 11). No significant differences emerged. Data are represented as means ± SEM and were analysed with unpaired *t*-tests. * *p* < 0.05; ns = not significant as *p* > 0.005.

A key finding of this study concerns the elevated psychological burden experienced by pregnant women who tested positive for COVID-19 (Figure 1). Specifically, results from the BDI indicated significantly higher levels of depressive symptoms among COVID-19-positive participants compared to those who tested negative (U = 27, *p* = 0.048). Interestingly, this group difference did not emerge in scores on the EPDS, suggesting that the emotional distress observed was not indicative of postnatal depression per se, but rather a response to the acute stress of infection and the broader context of the pandemic. Trait Anxiety scores were comparable between groups, indicating that the baseline predisposition to anxiety was not significantly impacted by COVID-19 status. However, State Anxiety scores—measured by the STAI—revealed moderate to high anxiety levels in both groups, with significantly higher levels among COVID-19-positive women (U = 27, *p* = 0.048; Figure 1). This highlights the acute, situational nature of their distress and illustrates how the experience of illness, hospitalisation, and concern for foetal outcomes can markedly amplify emotional reactivity during pregnancy [14,17,24].

### 3.3. Trauma-Related Stress and Emotional Strain

**Figure 2:**
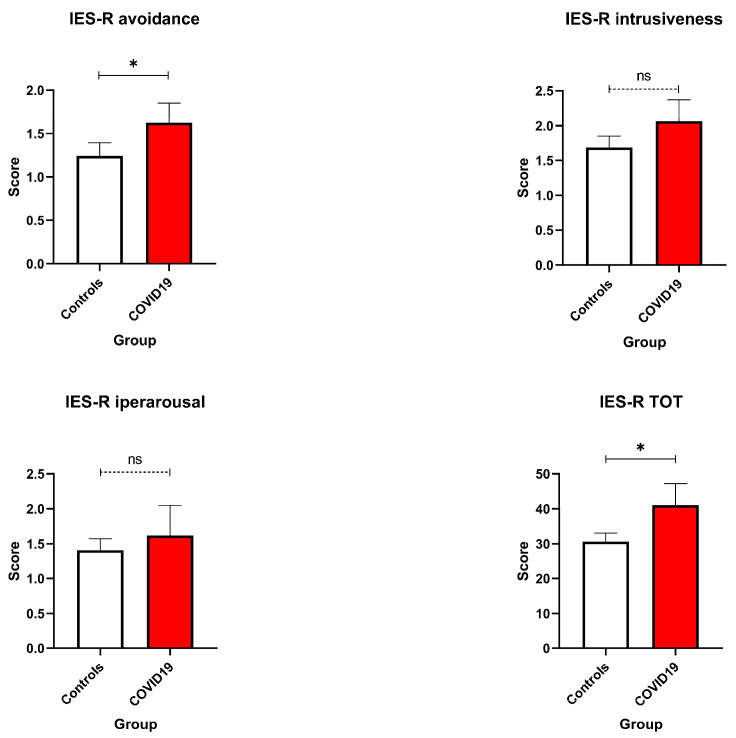
IES-R scores. Avoidance, intrusiveness, hyperarousal, and Total Score were compared between COVID-19 pregnant women (red—N = 10) and the control ones (white—N = 11). Data are represented as means ± SEM and were analysed with unpaired *t*-tests. * *p* < 0.05; ns = not significant as *p* > 0.005.

The psychological impact of COVID-19 infection during pregnancy was further evident in trauma-related outcomes. On the IES-R, COVID-19-positive women reported significantly higher total scores (U = 24.5, *p* = 0.029) and increased avoidance behaviours (IES-R Avoidance: U = 26, *p* = 0.036; Figure 2).

These results point to elevated post-traumatic stress symptoms, particularly in the form of emotional detachment and avoidance-related coping mechanisms, which may reflect an effort to manage overwhelming stress related to health fears and isolation [25]. While hyperarousal symptoms, such as irritability or difficulty sleeping, did not differ significantly between groups, the elevated IES-R scores suggest that infection during pregnancy can constitute a psychologically traumatic experience with potential long-term implications.

### 3.4. Physical Functioning and Health Perceptions

**Figure 3:**
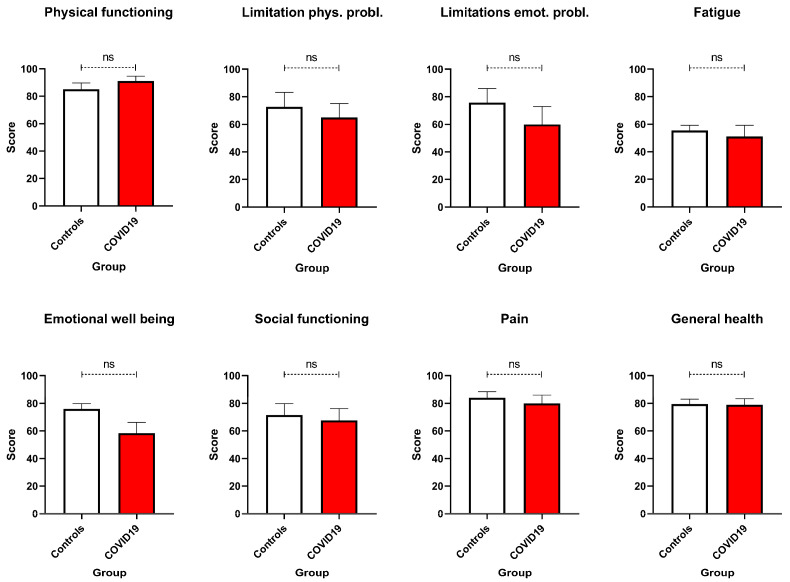
SF-36 scores. Physical functioning, limitations due to physical and emotional problems, fatigue, emotional well-being, social functioning, pain, and general health were compared between COVID-19 pregnant women (red—N = 10) and the control ones (white—N = 11). No significant differences emerged. Data are represented as means ± SEM and were analysed with unpaired *t*-tests. ns = not significant as *p* > 0.005.

In contrast to mental health outcomes, physical health remained largely stable across both groups. SF-36 Physical Functioning scores were similar, with average ratings of approximately 83% for both COVID-19-positive and COVID-19-negative participants (Figure 3).

Thus, despite heightened emotional distress, most women maintained good physical functioning during the perinatal period. Nonetheless, fatigue emerged as a prevalent concern in both groups, with average scores around 51.9%, reinforcing the importance of monitoring physical energy and recovery regardless of infection status. General health perceptions also did not differ significantly between groups. However, it is noteworthy that some COVID-19-positive women reported feeling physically unwell despite stable objective measures, suggesting that emotional distress may negatively influence subjective health appraisals.

### 3.5. Breastfeeding Experience and the Role of Social Support

Breastfeeding emerged as a particularly challenging aspect of the postpartum period. While a minority of participants described the experience as positive, the majority found it emotionally and physically demanding. These findings underscore the need to strengthen breastfeeding support services, particularly during crises, when external guidance and reassurance may be limited. Finally, the results highlight the importance of social determinants of health in moderating psychological outcomes. Both groups reported similar levels of spousal education, suggesting that partner support may have served as a protective factor during the pandemic. Additionally, smaller household sizes may have contributed to lower caregiving burdens, thereby reducing stress. Together, these findings emphasise that strong social support systems and socioeconomic stability play a critical role in promoting resilience among pregnant and postpartum women during public health emergencies [24,25,26].

## 4. Discussion

This exploratory pilot study investigated the psychological and physical health of postpartum women during the COVID-19 pandemic, uniquely conducted in Reggio Emilia—a province among the hardest hit in Italy during the early outbreak. Our study is distinguished by its focus on remote mental health assessments conducted between 6 and 12 months postpartum, offering valuable insights into the longer-term psychological impact beyond the immediate postpartum period. We also provided thorough clinical and demographic profiling of women with PCR-confirmed COVID-19 at childbirth compared to COVID-negative controls, allowing a nuanced understanding of how infection status relates to maternal mental health.

Consistent with these efforts, COVID-19-positive women showed significantly higher depressive symptoms on the BDI, reflecting the compounded psychological burden of infection-related anxiety, uncertainty about maternal–foetal outcomes, and social isolation [27]. In contrast, EPDS scores did not significantly differ between groups, with mean scores for both remaining below the clinical threshold of ≥13, indicating that postnatal depressive symptoms may not have been as pronounced as those during pregnancy. This divergence highlights a possible temporal decoupling of pandemic-related distress and postnatal emotional recovery, emphasising the need to consider contextual stressors beyond childbirth itself [4]. Anxiety-related findings revealed nuanced distinctions: STAI-T did not differ significantly between groups, suggesting similar baseline anxiety predispositions. However, STAI-S was significantly elevated in COVID-positive women, indicating acute, situational distress likely triggered by hospitalisation, infection, and fear for foetal health [28]. These findings underscore the relevance of state-level assessments during crises, which may be more sensitive to transient but clinically significant distress [14,17,24]. Post-traumatic stress symptoms, assessed via the IES-R, were also elevated in the COVID-positive group. Notably, avoidance subscale and total IES-R scores approached or exceeded the clinical concern threshold of ≥33, indicating potential risk for PTSD-like symptoms [29,30]. Physical functioning outcomes were generally comparable between groups. However, fatigue was a common complaint, likely reflecting both typical postpartum changes and emotional exhaustion associated with pandemic-related stressors. These findings support an integrative biopsychosocial model of maternal health, where mental and physical outcomes are deeply interwoven. Social determinants of health emerged as potential protective factors. Descriptive trends indicated that women with higher partner education levels or broader family support networks reported fewer psychological symptoms—consistent with prior research on the buffering effects of social support and socioeconomic stability [31,32].

Importantly, the remote data collection system was designed for assessment purposes only, without embedding formalised interventions such as scheduled counselling or lactation support. Although participants had access to standard clinical care, no structured psychosocial services were provided as part of the study protocol.

Broader conceptual insights emerged from this naturalistic, “wait-and-see” study design, which allowed observation of how women coped with the compounded stressors of pregnancy and a global health crisis. These observations highlight the potential value of real-time health screenings, early psychosocial interventions, and structured follow-up protocols in mitigating psychological harm.

While this exploratory pilot study provides meaningful insights into the psychological and physical health of postpartum women during the COVID-19 pandemic, several methodological limitations must be acknowledged, each of which also highlights critical opportunities for improvement in future research. First, the relatively small sample size limits statistical power and the generalisability of findings; however, it reflects the real-world barriers to recruitment and participation during a public health emergency. The stress and uncertainty of the pandemic, combined with concerns about privacy and medical fatigue, may have discouraged eligible women from enrolling, particularly those already burdened by psychological distress. Future studies should proactively address these barriers by embedding recruitment within routine clinical workflows, offering flexible digital participation options, and providing immediate support resources to reduce perceived burden. Second, the single-centre design and inclusion of only women with normal pregnancies may have constrained sample diversity and excluded individuals at higher clinical or social risk. Expanding to multicentre cohorts and including women with high-risk pregnancies or varied socio-demographic backgrounds would enhance both representativeness and the external validity of findings. Third, the study’s cross-sectional structure, while suited to an emergent situation, did not allow for tracking changes over time or establishing temporal relationships between stressors and outcomes. Future research should adopt longitudinal designs with multiple time points to better capture the evolving nature of perinatal psychological resilience and vulnerability. Finally, although no formal intervention was embedded within the remote assessment platform, the study demonstrates that telehealth-based screening is both feasible and acceptable to postpartum women, even in a crisis context.

Thus, despite these limitations, our pilot study also offers a roadmap for more robust, inclusive, and responsive maternal mental health research, and taught us important lessons for maternal mental health research and care delivery in the context of public health emergencies. One key lesson from this pilot is the importance of methodological transparency and adaptability. Our original intent to conduct longitudinal follow-up was hindered by real-world challenges: participant attrition, inconsistent digital access, and the psychological toll of the crisis itself. These obstacles, which also affected many larger pandemic-era studies [28,33], highlight the need for flexible, patient-centred retention strategies and more resilient research infrastructure for maternal mental health. Moreover, the study’s shift from a longitudinal to a cross-sectional framework was not merely a limitation: it reflected an important insight.

In particular, research in emergency contexts must be designed to accommodate uncertainty, while still upholding standards of data integrity and ethical care.

A second lesson is the value of using multiple validated instruments to capture different dimensions of distress. Our findings that BDI scores exceeded clinical thresholds while EPDS scores remained below suggest that broader depressive symptoms—rather than those strictly linked to childbirth—may dominate during crisis conditions. This distinction has been supported by other research, including large-scale studies in China and the U.S., which found elevated rates of general depressive symptoms during the pandemic, even when perinatal-specific tools like the EPDS failed to detect clinical severity [4,27]. This underscores the importance of choosing context-sensitive assessment tools and interpreting scores in light of external stressors.

Another lesson involves the relevance of remote monitoring platforms. Our study demonstrated that remote data collection is feasible for assessing psychological and physical health in postpartum women, even under constrained and rapidly evolving conditions. This is consistent with other evaluations of digital maternal health interventions during the pandemic, which found that app-based or telemedicine platforms could effectively monitor depression and anxiety symptoms [28,34]. Along with data collection we must remember the importance of establishing a targeted intervention, suggesting that future digital platforms should combine screening with proactive care pathways, such as real-time counselling, peer support groups and selected referrals to mental health professionals.

The study also reaffirmed the protective influence of social determinants, particularly family support and partner education level, on maternal mental health outcomes. Although our findings were descriptive, they align with decades of evidence showing that robust social networks and socioeconomic stability reduce perinatal psychological distress [32,33]. In contrast, women lacking such supports during the pandemic were disproportionately affected by depression, anxiety, and trauma-related symptoms [31,35]. Thus, policies and interventions aiming to strengthen perinatal mental health must address not only individual risk but also the broader social and structural determinants that shape maternal vulnerability and resilience. Importantly, this study contributed to reframing maternal mental health as a critical domain of emergency preparedness and response. The study’s naturalistic, “wait-and-see” design allowed for observation of unprompted coping responses, revealing both strengths—such as emotional recovery in some women by 12 months postpartum—and gaps, particularly in early support and structured follow-up [29,30]. These insights have since informed emerging guidelines on integrating maternal mental health screening into pandemic planning and telehealth expansion [34,36]. Finally, the study underscores the enduring value of pilot studies in advancing maternal mental health science, especially during crises. It also catalysed dialogue about remote assessment feasibility, tool selection, and the intersection of clinical care with digital infrastructure.

As healthcare systems continue to shift toward hybrid service models, the lessons from this pilot offer a foundation for designing scalable, equitable, and crisis-resilient maternal care systems [33,35,37].

Importantly, telehealth and digital tools, while efficient, may risk reduced engagement if not designed with empathy, trust-building, and ongoing contact. To bridge this gap, we propose a conceptual framework: “10 Gold Rules for Remote Medicine Support to Pregnant Women in Critical Situations” (Table 2). While not empirically validated within the scope of this study, these principles are expert-informed, drawing on the diverse and complementary expertise of our multidisciplinary team—including obstetricians, paediatricians, psychologists, pharmacologists, and neuroscientists. This collaborative foundation enabled the development of a holistic, patient-centred model that reflects both clinical realities and psychosocial needs. Moreover, our group has a strong track record in advocating for patient-centric approaches and building trust in clinical research, particularly in vulnerable populations. These principles are intended as a blueprint to guide the design of compassionate, adaptable, and scalable remote care systems—capable of maintaining continuity, equity, and emotional safety for pregnant women during future health emergencies [38,39,40].

## 5. Conclusions

Pregnancy and postpartum care require sustained, responsive support—particularly under the added stressors of public health emergencies. This exploratory pilot study sheds light on the psychological vulnerability of perinatal women during COVID-19 and highlights the viability of remote data collection as a research and monitoring tool. While the data do not support broad generalisations or causal claims, they offer a compelling rationale for deeper investigation into digitally supported maternal care. The proposed “10 Gold Rules” serve not as definitive guidance, but as a foundation for future inquiry—guiding the development of more robust, equitable, and responsive maternal health strategies during crises. As healthcare systems continue to evolve, especially in light of digital innovation, these preliminary concepts can inform adaptive interventions that prioritise maternal well-being, emotional resilience, and continuity of care beyond the pandemic.

## Figures and Tables

**Table 1:** Summary of the variables collected from pregnant women who contracted COVID-19 (study group) and who did not contract COVID-19 (control group). Means with their standard deviations, medians, Mann–Whitney U tests and their relative *p*-values are reported.

Continuous Variables	Control Group		COVID-19-Positive Group		Mann–Whitney U	*p*-Value
	Mean (SD)	Median	Mean	Median		

Age (years)	33 (4.1)	34	35.1 (4.33)	34	36.5	0.201
Education (years)	14.36 (1.57)	13	13.6 (1.26)	13	41	0.361
Husband’s Age (years)	32.6 (7.9)	35	37.8 (4.34)	34	30	0.078
Husband’s Education (years)	12 (3.41)	13	11.8 (2.78)	13	51.5	0.949
Family Members	2.5 (0.69)	3	3.11 (0.33)	3	\	\
Beck Depression Inventory—BDI	6.73 (4.73)	6	13.5 (8.14)	15	27	0.048 *
Edinburgh Postnatal Depression Scale	9.55 (6.69)	11	7.3 (5.85)	6.5	44.5	0.475
Beck Anxiety Inventory—BAI	7.73 (6.23)	7	10.6 (8)	8	45	0.498
STAI-State Anxiety	33.64 (10.15)	32	41.6 (10.23)	39.5	27	0.048 *
STAI-Trait Anxiety	36.18 (6.55)	35	44.8 (10.92)	44	30	0.081
IES-R Avoidance	1.24 (0.51)	1.38	1.63 (0.71)	1.57	26	0.036 *
IES-R Intrusiveness	1.68 (0.54)	1.88	2.07 (0.97))	2.19	36.5	0.198
IES-R Hyperarousal	1.41 (0.54)	1.33	1.62 (1.97)	1.5	54.5	0.985
IES-R Total Score	30.64 (7.99)	31	41.1 (19.33)	38	24.5	0.029 *
Physical Functioning (SF-36)	85 (15)	90	91 (11.74)	95	39	0.261
Limitation due to Physical Problems (SF-36)	72.73 (34.38)	100	65 (31.62)	62.5	47	0.6
Limitation Due to Emotional Problems (SF-36)	75.75 (33.65)	100	59.98 (40.98)	66.6	42	0.346
Fatigue (SF-36)	55.45 (12.93)	60	51 (26.12)	40	47	0.59
Emotional Well-Being (SF-36)	76 (12.77)	80	58.4 (24.53)	64	30	0.079
Social Functioning (SF-36)	71.59 (27.44)	87.5	67.5 (27.76)	68.75	51	0.794
Pain (SF-36)	84.09 (14.55)	90	80 (19.22)	78.75	50.5	0.763
General Health (SF-36)	79.55 (11.5)	80	79 (13.7)	85	54	0.965

Dichotomic variables	YES (%)	NO (%)	YES (%)	NO (%)	\	\

First Pregnancy	7 (63.6)	4 (36.4)	8 (80)	2 (20)	\	\
Anxiety During Pregnancy	3 (27.3)	8 (72.7)	4 (40)	6 (60)	\	\
Anxiety after pregnancy	6 54.5)	5 (45.5)	9 (90)	1 (10)	\	\
Scared of the future	10 (90.9)	1 (9.1)	9 (90)	1 (10)	\	\
Difficulties during Breastfeeding	6 (54.5)	5 (45.5)	4 (40)	6 (60)	\	\
Support from clinicians	4 (36.4)	7 (63.6)	6 (60)	4 (40)	\	\
Breastfeeding is positive for your wealth	6 (54.5)	5 (45.5)	7 (70)	3 (30)	\	\
Depression after pregnancy	2 (18.2)	9 (81.8)	4 (40)	6 (60)	\	\

Asterisks indicate statistical significance. * *p* < 0.05.

**Table 2:** 10 Gold Rules for Remote Medicine Support to Pregnant Women in Critical Situations.

Rule	Explanation	Rationale	Implementation
**Communicate Clearly and Accessibly**	Use plain, culturally sensitive language; ensure platforms are intuitive and mobile-friendly.	Miscommunication increases anxiety and reduces trust; pregnant women with low health literacy or language barriers need clear, actionable info.	Provide translated materials, visual aids, and voice-overs. Keep instructions simple (e.g., appointment scheduling, medication use). Use platforms requiring minimal technical skills.
**Prioritise Mental Health Support**	Routinely screen for stress, anxiety, and depression with validated tools (EPDS, GAD-7, PHQ-9); offer referrals or digital interventions.	Maternal mental health can deteriorate rapidly in crises; early detection prevents escalation.	Embed screening in remote check-ins or patient portals. Offer tele-psychology, mindfulness apps, and crisis counsellor referrals.
**Deliver Personalised Care Plans**	Tailor care to medical history, pregnancy stage, cultural context, and psychosocial needs.	One-size-fits-all care is ineffective during crises with variable stressors and resources.	Use intake forms to collect relevant data. Adapt follow-ups (e.g., anxiety management for high-risk, breastfeeding support). Provide flexible scheduling.
**Leverage Remote Monitoring Technologies**	Use digital tools (blood pressure cuffs, foetal monitors, surveys) to reduce hospital visits.	Minimises infection risk and travel burden, critical when mobility/access is limited.	Distribute monitoring kits. Use smartphone-compatible platforms to track vitals and mental health, with alerts for abnormal readings.
**Provide Continuous Virtual Lactation Support**	Offer real-time or asynchronous virtual consultations with lactation specialists.	Breastfeeding challenges cause distress if unaddressed; in-person help may be unavailable.	Schedule video consultations postpartum. Provide instructional videos and 24/7 chat for urgent issues like latching or milk supply.
**Encourage Family Involvement**	Engage partners and family in care discussions and decisions where appropriate.	Strong social support protects against perinatal mental health disorders and reduces isolation.	Invite family to telehealth visits. Provide educational materials tailored for caregivers.
**Safeguard Privacy and Data Security**	Ensure compliance with privacy laws (HIPAA, GDPR); protect data and communications.	Trust depends on confidentiality; privacy breaches reduce participation.	Use encrypted platforms, two-factor authentication, and informed consent. Be transparent about data use and access.
**Give Timely, Clear Emergency Guidance**	Provide clear instructions on warning signs and when to seek in-person care.	Remote care risks delays unless women know critical red flags.	Use checklists, videos, colour-coded symptom trackers. Include “click-to-call” emergency contact options within platforms.
**Ensure Consistent Follow-Ups**	Schedule regular virtual check-ins (e.g., every 1–2 weeks) to monitor and update care.	Prevents missed warning signs, loss to follow-up, and disengagement; fosters connection.	Automate reminders, use chatbots for low-risk follow-ups, escalate to human contact when needed.
**Promote Social Support Networks**	Facilitate access to peer groups, community resources, and support networks tailored to pregnancy and parenting.	Shared experiences reduce isolation and empower women through mutual support.	Create moderated online forums or chat groups (e.g., first-time mothers, high-risk pregnancies). Share info on local services (food assistance, counselling, childcare).

## Data Availability

The datasets generated and analysed during the current study are not publicly available. Researchers interested in accessing anonymised data for justified academic purposes may submit a formal request to the corresponding authors.
